# Graphene Assisted in the Analysis of Coumarins in Angelicae Pubescentis Radix by Dispersive Liquid–Liquid Microextraction Combined with ^1^H-qNMR

**DOI:** 10.3390/molecules26092416

**Published:** 2021-04-21

**Authors:** Yanmei Feng, Qian Li, Daiyu Qiu, Guichen Li

**Affiliations:** Gansu Provincial Key Laboratory of Aridland Crop Science, College of Agronomy, Gansu Agricultural University, Lanzhou 730070, China; fym15693446892@163.com (Y.F.); qiudy@gsau.edu.cn (D.Q.); lguchen@163.com (G.L.)

**Keywords:** DLLME-SFO, graphene, ^1^H-qNMR, APR, coumarin

## Abstract

The content of active components in traditional Chinese medicine is relatively small, and it is difficult to detect some trace components with modern analytical instruments, so good pretreatment and extraction are very important in the experiment. Graphene was introduced by a dispersive liquid–liquid microextraction method based on solidification of floating organic drop (DLLME-SFO) with graphene/1-dodecyl alcohol used as the extractant, and this method, combined with quantitative proton nuclear magnetic resonance spectroscopy (^1^H-qNMR), was used to simultaneously qualitative and quantitative osthole, columbianadin and isoimperatorin in Angelicae Pubescentis Radix. In this experiment, a magnetic stirrer was used for extraction, all NMR spectra were recorded on a Bruker Advance III 600 MHz spectrometer with dimethyl sulfoxide-d_6_ (DMSO-d_6_) as deuterated solvent and pyrazine as the internal standard. The influencing factors and NMR parameters in the extraction process were investigated and optimized. In addition, the methodology of the established method was also examined. The quantitative signals of osthole, columbianadin and isoimperatorin were at a chemical shift of δ6.25–δ6.26 ppm, δ6.83–δ6.85 ppm, and δ6.31–δ6.32 ppm. The linear ranges of osthole, columbianadin and isoimperatorin were all 0.0455–2.2727 mg/mL, and R^2^ were 0.9994, 0.9994 and 0.9995, respectively. The limits of detection of osthole, columbianadin and isoimperatorin were 0.0660, 0.0720, 0.0620 mg, and the limits of quantification of osthole, columbianadin and isoimperatorin were 0.2201, 0.2401, 0.2066 mg/mL. The solution had good stability and repeatability within 24 h. The recoveries of osthole, columbianadin and isoimperatorin were 102.26%, 99.89%, 103.28%, respectively. The established method is simple and easy to operate, which greatly reduces the cumbersome pretreatment of samples and has high extraction efficiency.

## 1. Introduction

Angelicae Pubescentis Radix (APR) is the dried root of Angelicae pubescentis maxim f. biserrata Shan et Yuan in China [1]. In addition to a long medical history in China, it is also distributed in Korea and Japan [2]. APR is pungent and mild, and has a good effect when treating rheumatic arthralgia. From the perspective of the basic theory of traditional Chinese medicine (TCM), dampness evil is recognized as one of the main pathogenic factors of the coronavirus disease 2019. APR as monarch drug or adjuvant can dispel pathogenic windage, clear dampness and activate yang qi, and has played an important role in the outbreak [3,4]. Coumarin is a type of aromatic family of compounds with benzo-α-pyranone as the parent nucleus, which has been proved to be the compound with the most abundant content and the strongest biological activity [5]. In the 2020 edition of the Chinese pharmacopeia, coumarin components are regarded as one of the indexes to evaluate its quality. In modern pharmacological studies, coumarin has anti-inflammatory and analgesic [6,7], rheumatoid arthritis [8], anti-tumor [9], and anti-bone marrow osteoporosis effects [10]. 

In short, a dispersive liquid–liquid microextraction (DLLME) is a liquid phase microextraction technique [11]. During the DLLME development era, myriad of innovations were introduced [12,13,14,15]. Among them, dispersive liquid–liquid microextraction based on the solidification of floating organic drop (DLLME-SFO) was proposed by Leong [16] in 2008, which was based on dispersive liquid–liquid microextraction (DLLME) [17,18,19,20] and liquid-phase microextraction based on solidification of floating organic drop (LLME-SFO) [21]. The extraction process is composed of a dispersant, extractant and sample solution, among which the dispersant is the key to form the emulsion. Its function is to disperse the extractant into small droplets in the solution and increase the contact area with the sample solution. Therefore, the purpose of dispersants with both hydrophilicity and lipophilicity is to be soluble in the extractant and sample solution simultaneously, such as methanol, ethanol, acetonitrile, etc. In addition, the extractant is not mutually soluble with water and should be nonvolatile, with a density less than that of water and a melting point close to room temperature, so as to float on the sample solution and form suspended droplets, which would be convenient in experimental operation. Commonly used extractants include dodecyl alcohol, 2-dodecyl, undecyl and some halogenated hydrocarbons. In summary, Leong combined the advantages of the above two methods and overcame their disadvantages: toxic extraction solvents were needed in DLLME, such as chlorobenzene, chloroform and carbon tetrachloride, which were not conducive to human health for a long time; in LLME-SFO, although the amount of organic solvent in the extraction process is reduced, the extraction speed is slow. Compared with the above methods, DLLME-SFO extraction process is simple, more efficient and more environmentally friendly. The methods are currently used for determination of traces of lead and Hg^2+^ in aqueous samples [22,23], pesticide residues and heavy metals [24,25,26] in food and trace amounts of aflatoxin [27] in spoiled food. It was also used for pharmacological studies [28] in medicine. In practical applications, DLLME-SFO is generally combined with modern analytical instruments, such as high-performance liquid chromatography (HPLC) [29,30,31], gas chromatography (GC) [32,33,34], and atomic absorption spectroscopy (AAS) [35,36,37], etc. However, this method has not been reported to be combined with nuclear magnetic resonance spectrometer (NMR), and it would been used for the first time for the qualitative and quantitative analysis of the osthole (OS), columbianadin (CO) and isoimperatorin (IS).

Graphene is a two-dimensional flat monolayer structure composed of carbon atoms connected by sp_2_ hybridization with a honeycomb shape [38]. It has good hardness, electrical and thermal conductivity, it also has the property of reconciling the contradiction between brittleness and ductility [39,40]. In addition to the above characteristics, graphene has strong adsorption capacity due to the presence of a large number of carboxyl, hydroxyl and epoxy groups on the surface. At present, many graphene-based extraction methods have been successfully established [41,42,43]. In the previous studies, it was found [44] that there are unique advantages in the determination of the effective components in TCM by quantitative proton nuclear magnetic resonance spectroscopy (^1^H-qNMR). (1) There is no special requirement for the determination of compounds. (2) There is no damage to the analyte and it can be recovered after the measurement. (3) A large number of reference substances are not needed, and multiple indicators can be determined simultaneously only by selecting appropriate internal standard substances and the deuterium-substituted solvent, which is more consistent with the diversity of effective components in TCM. However, there are some problems such as low sensitivity and strong interference when qNMR is used for extremely low concentration and trace substances, so the sample needs to be enriched or concentrated in the early treatment. DLLME-SFO, as a pre-treatment method that can be integrated with extraction and concentration, has high extraction efficiency, high speed and simple operation. Therefore, DLLME-SFO was combined with ^1^H-qNMR, graphene was introduced [45] to increase the adsorption and extraction efficiency and graphene/1-dodecyl alcohol was used as extraction agent, which was helpful to solve the problem of low concentration and strong interference (Table 1). This method was used for the first time to quantitatively and qualitatively analyze the OS, CO and IS in APR.

## 2. Results and Discussion

### 2.1. Optimization of NMR Parameters

NS is the number of repeated samples. If NS is too small, insufficient scanning will result in poor accuracy and reproducibility; when NS is set high, the reproducibility is better and the signal-to-noise ratio can be improved to make the baseline of spectrogram more stable and flat, but at the same time the detection time will be prolonged. The process of the nucleus from the excited state to the equilibrium arrangement state is called the relaxation process, this process is a necessary condition for the generation of nuclear magnetic resonance phenomenon, the time required by it is called D1. In the ^1^H-qNMR experiment, the appropriate D1 directly depends on the accuracy of quantitative results, and the suitable D1 can ensure the complete relaxation of the quantitative nucleus and the spectrum peak will not be saturated, which is conducive to the correct integration of the quantitative peak. If the D1 is too small, the quantitative results will be inaccurate and distorted, but D1 should not be set too high which would cause the extension of detection time.

In this experiment, D1 and NS were set step by step to accumulate multiple experiments, and samples were taken from small to large according to D1 and NS. Then, the quantitative characteristic peaks of analytes and internal standard (pyazine) were integrated, and D1 and NS values whose integral area ratio no longer changed significantly were selected.

#### 2.1.1. Choice of D1

Through the investigation of different D1 (1, 5, 15, 20, 25, 30, 40, 50, 70, 90 and 100 s), the results showed that, with the gradual increase of D1, detection analysis time was also extended and the integral area was gradually decreased, and, when D1 was 15 s, the integral area no longer changed. After comprehensive consideration, we chose 15 s for D1.

#### 2.1.2. Choice of NS

When NS was 16, 32, 64 and 128 times, the influence on the integral area was investigated. According to the analysis results, NS has no influence on the integral area, and with the increase of NS, the detection and analysis time were also extended, so NS was set at 16 times.

### 2.2. Optimization of DLLME-SFO Conditions

The optimization of DLLME-SFO conditions were obtained by investigating the volume ratio of ultrapure water to the sample solution, the types, concentration and volume of extractant, different ionic strength, different pH, different speed and different extraction time. The enrichment factor (ER) is defined as the ratio of the total amount of the measured substance in the extraction phase to the initial amount of the measured substance in the sample phase. The specific calculation formula is as follows:

$$\mathrm{ER}=\frac{100\times V_{0}C_{0}}{V_{s}C_{s}+V_{0}C_{0}}$$

C_0_ and Cs are the concentration of the substance to be measured in the extraction solvent phase and the sample phase when the extraction reaches equilibrium. V_0_ and Vs. are the volume of the extraction solvent phase and the sample phase respectively.

#### 2.2.1. Choice of Extractant and Dispersant

**Figure 1:**
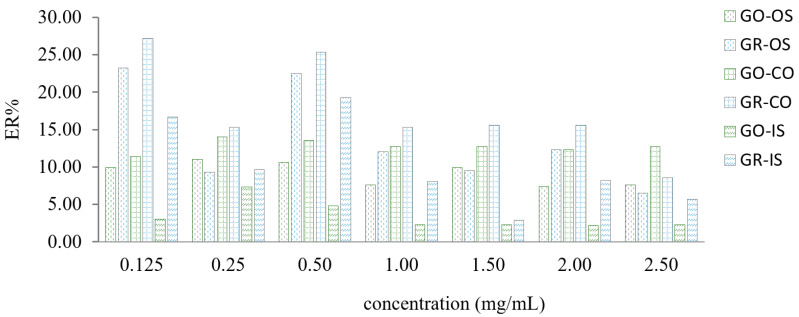
Choice of extractant. Note: graphene oxide, GO; graphene, GR.

In the experiment, graphene and graphene oxide were selected to investigate the effects on ER of OS, CO and IS within the range of 2.5–0.125 mg/mL, respectively. As can be seen from Figure 1, the extraction effect of graphene was better; in this study, methanol was used to extract medicinal materials. Therefore, in order not to introduce other dispersion items to interfere with the extraction solution, methanol was used as a dispersant during the experiment.

#### 2.2.2. Selection of Volume Ratio of Distilled Water to Test Sample Solution

When the volume ratio of distilled water to the test solution was 4:6, 5:5 and 6:4, the solvent after extraction was a white, turbid liquid, and the turbidity degree was more pronounced with no large droplets appearing. When the volume ratio was 7:3, the solvent after extraction was still a white, turbid liquid, although the turbidity degree was relatively low and there was also no large droplets. When the volume ratio was 8:2 and 9:1, there was no turbidity and droplets appeared in the extracted solution. ER was calculated and compared according to the experimental results. Finally when the volume ratio of distilled water to the test solution was 9:1, the extracted solution had no turbidity and the extraction rate was the highest.

#### 2.2.3. Selection of Graphene Concentration in 1-Dodecyl Alcohol

**Figure 2:**
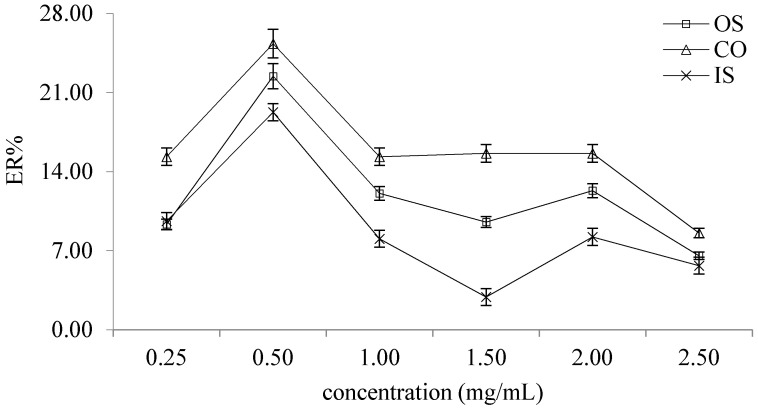
The influence of the concentration of graphene in 1-dodecyl alcohol on ER of OS, CO and IS.

The study investigated the influence of the concentration of graphene in 1-dodecyl alcohol at 2.5–0.25 mg/mL on ER. It can be seen from the Figure 2 that when the concentration of graphene in 1-dodecyl alcohol was 0.50 mg/mL, the ER of OS, CO and IS were the highest.

#### 2.2.4. Selection of Extractant Volume

**Figure 3:**
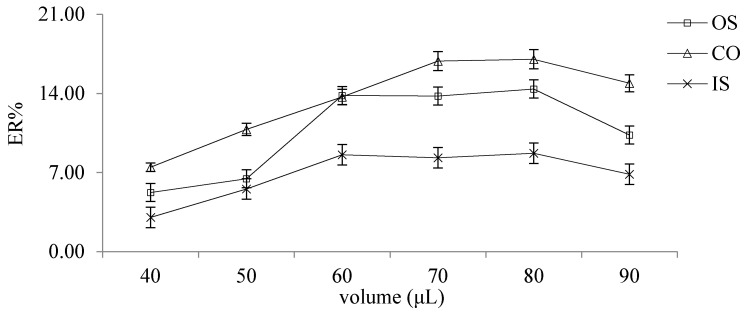
The effect of the volume of the extractant on ER.

In order to evaluate the effect of the volume of the extractant on ER, different volumes of graphene/1-dodecyl alcohol (40, 50, 60, 70, 80 and 90 μL) were selected for investigation. As shown in the Figure 3, the extraction efficiency was the highest when the volume was 80 μL.

#### 2.2.5. Selection of Extractant Volume

**Figure 4:**
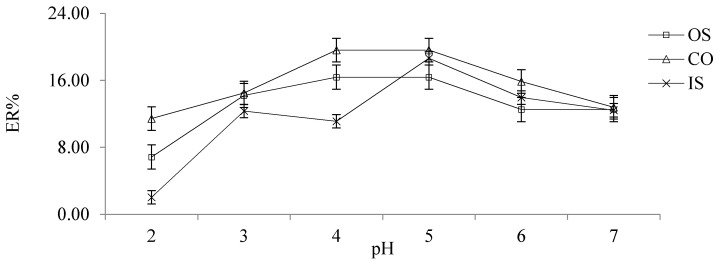
The effect of pH on ER.

The lactones in coumarin components are prone to ring opening and hydrolysis under alkaline conditions. Therefore, the effect of pH on ER at 2–7 was analyzed by adding 0.40 mol/L HCl and 0.40 mol/L NaOH. When the pH of the solution was 5, the ER of OS, CO and IS were the highest, at 16.39%, 19.62% and 11.11%, respectively. The detailed results are shown in Figure 4.

#### 2.2.6. Choice of Ionic Strength

**Figure 5:**
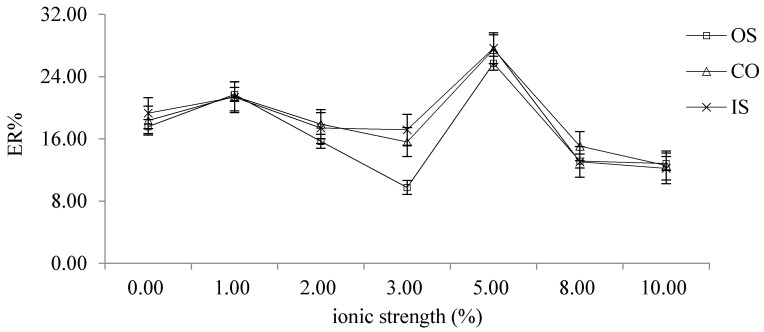
The effects of different ionic strength on ER.

The effects of different ionic strength (0%, 1%, 2%, 2.5%, 5%, 8% and 10%) on the extraction efficiency were investigated by adding different concentrations of NaCl into the extraction solution. The results showed that when NaCl concentration was 5%, the ER% of OS, CO and IS was the best. (Figure 5)

#### 2.2.7. Selection of Different Speed

**Figure 6:**
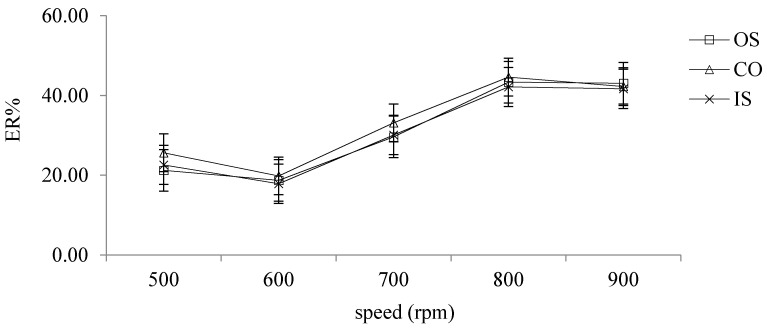
The effects of different speed on ER.

The effect of speed on extraction efficiency was investigated by setting the speed to 500, 600, 700, 800, 900, 1000, 1200 and 1400 rpm. The experimental results showed that, when the speed was greater than or equal to 1000 rpm, the extracted solution presented white turbidity without droplets, and as can be seen from Figure 6, when the speed reached 800 rpm, the ER of the target analytes gradually tended to be stable and reached the highest point, so 800 rpm was chosen as the speed.

#### 2.2.8. Different Extraction Times

**Figure 7:**
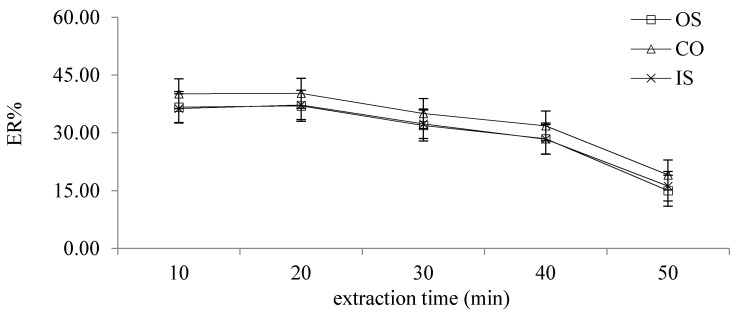
The effects of different extraction times on ER.

The extraction time was the period from the addition of the magnetic rotor into the extraction solution to the end of extraction. The effects of different extraction times (10, 20, 30, 40 and 50 min) on ER were investigated. As can be seen from the Figure 7, when the extraction time was 10–20 min, the ER of the target analyte gradually increased and reached its maximum at 20 min. When the extraction time was more than 20 min, ER decreased with the extension of the extraction time. Therefore, 20 min was selected as the extraction time in this study.

### 2.3. Methodology Validation

#### 2.3.1. Linearity

The appropriate amount of OS, CO and IS was precisely weighed and prepared into a mixed solution, then diluted to eight different concentrations. The linear range of OS, CO and IS were all 0.0455–2.2727 mg/mL, and R^2^ were 0.9994, 0.9994 and 0.9995, respectively. The results showed that there was a good linear relationship between the internal area and the concentration (Table 2).

#### 2.3.2. Limit of Detection and Limit of Quantification

Limit of detection (LOD) is the smallest amount that the method can reliably detect to determine presence or absence of an analyte. Limit of quantification (LOQ) is the smallest amount that the method can reliably measure quantitatively to an acceptable level. Using the equation X_B_ + kσ_B_, where X_B_ and σ_B_ are the mean and the standard deviation of blank measurements, and k is the numerical factor determining the confidence level, LOD: k = 3, LOQ: k = 10 [46]. The LOD and LOQ of OS, CO and IS were analyzed as shown in the Table 2 below.

#### 2.3.3. Precision

High, medium and low concentrations were selected from the mixed reference materials and each concentration was measured in parallel for three times to determine precision. The RSD of OS, CO and IS were 1.10%, 1.07% and 0.90%, respectively, indicating that the precision of the instrument was good.

#### 2.3.4. Stability and Repeatability

In order to monitor the stability of the extracted sample solution, the same batch of medicinal materials should be measured under the same experimental conditions at 0, 2, 4, 8, 12 and 24 h. The RSD of OS, CO and IS were 1.56%, 1.04% and 1.54%, respectively, indicating that the samples were relatively stable within 24 h. The same batch of samples was repeated in order to investigate the repeatability of the established method, the RSD of OS, CO and IS were 2.56%, 2.13% and 3.70%, respectively.

#### 2.3.5. Recovery

One milligram of APR powder with known content was precisely weighed, and an appropriate amount of reference substance was added. The powder was for detection and analysis which was extracted according to 2.1, and then extracted according to 2.2. The experimental results were shown in Table 3.

### 2.4. Determination of the Samples

The established method was used to determine OS, CO and IS of APR from different producing areas. The detailed results were shown in Table 4. The quantitative signal of OS was at δ6.25–δ6.26 ppm, CO was at δ6.83–δ6.85 ppm, and IS was at δ6.31–δ6.32 ppm.

## 3. Materials and Methods

### 3.1. Reagent

Nuclear magnetic resonance spectrometer (Bruker Advance III 600-MHz, Karlsruhe, Germany); magnetic stirrer (INNOTEG-Science One MR 1, Staufen, Germany); PS-30 ultrasonic cleaning machine (Shenzhen Dekang Science and Technology Co., Ltd., Shenzhen, China); HX203 telectronic balance (Fujian Huazhi Scientific Instrument Co., Ltd., Fujian, China); JFSD-100 disintegrator (Shandong Jingcheng Medical Equipment Manufacturing Co., Ltd., Shandong, China).

### 3.2. Chemical

**Figure 8:**
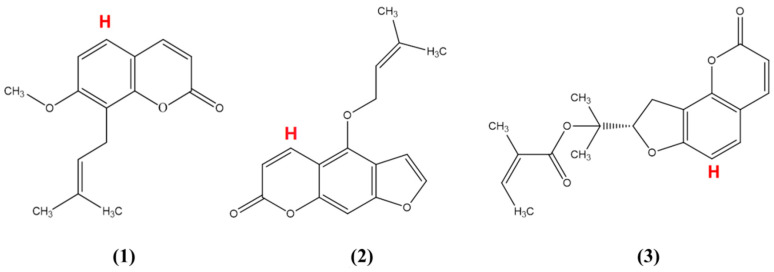
The chemical structural formulas of OS (1), CO (2), and IS (3).

Osthol (Lot EH150063, 98%), dimethyl sulfoxide-d_6_ (DMSO-d_6_) (99.9%) and pyrazine (98%) were all bought from Shanghai Energy Chemical Co., Ltd. (Shanghai, China), isoimperatorin (Lot 18062202, 98%) and columbianadin (98%) were purchased from Chengdu Pufei De Biotech Co., Ltd. (Chengdu, China), the chemical structural formulas were showed in Figure 8. Graphene and graphene oxide were all bought from Suzhou UG (Suzhou, China). Nano Material Co., Ltd. Methanol (≥99.5%) was bought from Tianjin Fuyu Chemical Co., Ltd. (Tianjin, China); 1-dodecyl alcohol (≥98%) was purchased from Shanghai Macklin Biochemical Co., Ltd. (Shanghai, China )NaCl (≥96%) was bought from Shanghai Sinopharm Chemical Reagent Co., Ltd. (Shanghai, China); HCl (≥96%) and NaOH (≥96%) were all bought from Tianjin Damao Chemical Reagent Factory (Tianjin, China).

### 3.3. Method

#### 3.3.1. Preparation of Sample Solution

APR was crushed and sifted, and 5 g of medicinal powder was precisely weighed and added with 50 mL methanol, which was extracted by ultrasonic (40 °C, 40 min), methanol was used to make up the missing weight, the extraction was filtered by decompression and stored at 4 °C in the dark for later use.

#### 3.3.2. Extraction Process of DLLME-SFO

**Figure 9:**
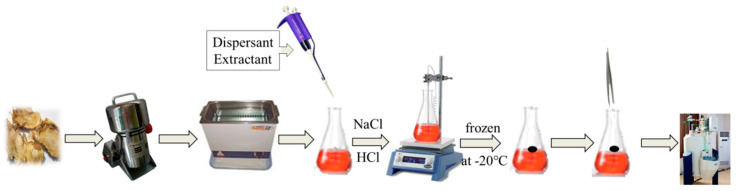
The specific experimental operation process.

**Figure 10:**
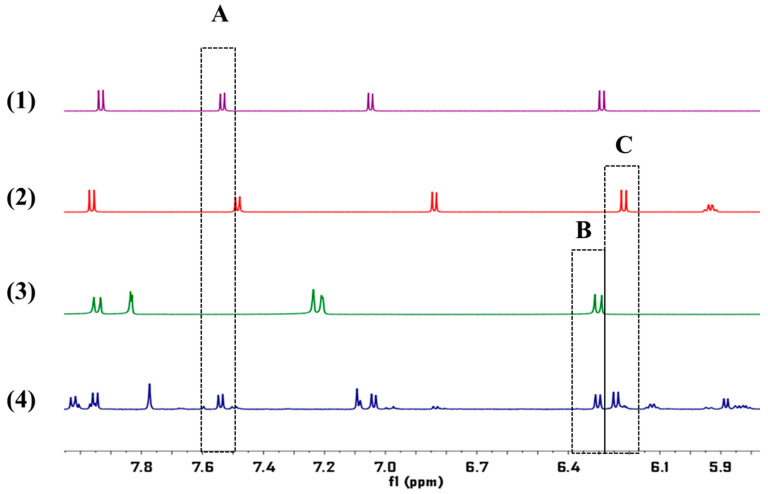
The ^1^H-NMR spectrum of OS (1), CO (2), IS (3) and APR (4) obtained at 600 MHz in DMSO-d6 solvent. The quantitative signals of OS (A), CO (B) and IS (C) were at chemical shift of δ6.25–δ6.26 ppm, δ6.83–δ6.85 ppm, δ6.31–δ6.32 ppm.

**Figure 11:**
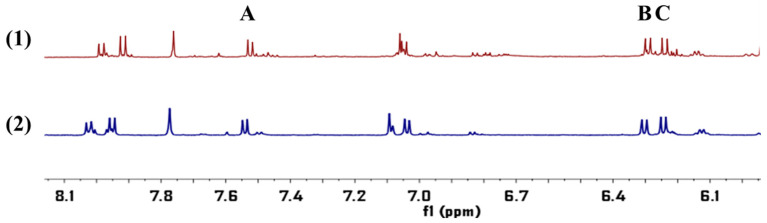
The ^1^H-NMR spectrum of APR: unextracted (1) and extracted (2), obtained at 600 MHz in DMSO-d_6_ solvent. The quantitative signals of OS (A), CO (B) and IS (C) were at chemical shift of δ6.25–δ6.26 ppm, δ6.83–δ6.85 ppm, δ6.31–δ6.32 ppm.

Graphene (2.5 mg) was precisely weighed and placed in a 10 mL volumetric flask. 1-dodecyl alcohol was added and ultrasonic was used for 1 h to make graphene completely dissolved in 1-dodecyl alcohol. After standing the solution was not stratified or precipitated, then graphene/1-dodecyl alcohol extractant was obtained, and the appropriate amount of the prepared extractant was absorbed and diluted to 0.5 mg/mL. The conical flask was fixed on the magnetic agitator, 9 mL ultrapure water and 1 mL test solution, 80 μL graphene/1-dodecyl alcohol (5 mg/mL) and 100 μL NaCl (5%) solution were added respectively, and the pH of the solution was adjusted to 5. Magnetic rotor was introduced in the conical flask, then extracted after 20 min (800 rpm, 40 °C) and let stand for 5 min, the conical flask was gently turned to form a large droplet, which was quickly frozen at −20 °C for 10 min. After solidification, the conical flask was removed with a small spoon and melted at room temperature, the accumulated phase was extracted with microinjection needle, besides the volume of accumulated phase was recorded and injected into the nuclear magnetic tube. Then 450 μL DMSO-d_6_ and 25 μL pyrazine (10 mg/mL) were added. The specific experimental operation process was shown in the Figure 9. The ^1^H-NMR spectrum of APR was shown in Figure 10. The preconcentration factor was obtained by dividing the concentration after extraction by the concentration before extraction. The preconcentration factors for OS, CO and IS were 270.01%, 248.30% and 268.29%, respectively. Although the preconcentration factors were low, they were useful for the QNMR method. It was found that the impurity peaks in the ^1^H-NMR spectrum were less and the separation degree was better after extraction (Figure 11).

#### 3.3.3. NMR Acquisition and Processing Parameters

All NMR spectra were recorded on a Bruker Advance III 600 MHz spectrometer. The spectrometer was operating at 600.10 MHz for 1H resonances, using a multinuclear broadband 5 mm probe. DMSO was chosen as deuterated solvent because of its superior solubility, preventing the solvent peak from overlapping with other peaks. Pyrazine was selected as the internal standard. Usual procedures for locking and shimming were conducted and followed by a careful tuning and matching adjustment for each sample. The pulse sequence was zg30, pulse width (P1) of 10.00 μs, test temperature (TE) of 295.6 K, spectrum width (SWH) of 11904.762 Hz, acquisition time (AQ) of 2.75 s, relaxation delay time (D1) of 1 s, scan times (NS) of 32 times.

## 4. Conclusions

The purpose of this study was to establish DLLME-SFO method, and graphene/1-dodecyl alcohol as extractant combined with ^1^H-qNMR for the first time to analyze OS, CO and IS in APR qualitatively and quantitatively. The extraction conditions were all optimized to obtain the best extraction method. The accuracy, stability and recovery of the established method were good after the methodology verification. Compared with other conventional methods, this method is more environmentally friendly, and has a high extraction efficiency, simple operation and good precision. The introduction of nanomaterials enhanced the adsorption and extraction, which help to solve the problem of low sensitivity and strong interference. It is worth discussing and explore for the extraction of effective components of TCM. It is hoped that this method can be used in the extraction of some valuable and rare effective ingredients in TCM in the future, which have special curative effect for some hard to treat diseases, such as cancer, HIV and so on.

## Figures and Tables

**Table 1:** The advantages of the established method compared with other methods.

Detection Method			Extraction Method		
	^1^H-qNMR	HPLC		DLLME-SFO	Other extraction methods
Requirement on sample form	No specific requirements	Liquid	Volume of samples required	1 mL	≥10 mL
Demand for reference substances	No	Yes	Need to stand layer	No	Yes
Detection test	≤5 min	>10 min	Extraction time	20 min	>20 min
Recycling of samples after measurement	Yes	No	Simultaneous separation and extraction	Yes	No

**Table 2:** Linearity, limit of detection and limit of quantification of OS, CO and IS.

Analyte	Linear Regression Equation	R^2^	LOD (mg/mL)	LOQ (mg/mL)
OS	y = 0.0798x + 0.0008	0.9994	0.0660	0.2201
CO	y = 0.0822x + 0.0007	0.9994	0.0720	0.2401
IS	y = 0.0784x − 0.0011	0.9995	0.0620	0.2066

**Table 3:** The recovery of OS, CO and IS.

Analyte	Original Content (g)	Added Content (g)	Total Observed (g)	Recovery (%)	Average Recovery (%)	RSD (%)
OS	0.0057	0.0057	0.0113	98.19	102.26	3.62
			0.0117	104.46		
			0.0117	106.12		
			0.0112	97.37		
			0.0117	105.13		
CO	0.0085	0.0085	0.0170	100.07	99.89	3.86
			0.0168	97.66		
			0.0166	94.29		
			0.0172	101.64		
			0.0175	105.79		
IS	0.0058	0.0057	0.0114	97.93	103.28	4.15
			0.0121	109.33		
			0.0118	103.68		
			0.0115	99.11		
			0.0119	106.34		

**Table 4:** The content of OS, CO and IS of APR from different producing areas.

Number	Content (g/g)		
	OS	CO	IS
1	0.0023	0.0040	0.0017
2	0.0016	0.0021	-
3	0.0024	0.0040	-
4	0.0003	0.0006	0.0007
5	0.0002	0.0010	0.0002
6	0.0026	0.0049	0.0030
7	0.0043	0.0061	0.0002
8	0.0042	0.0069	0.0007
9	0.0042	0.0069	0.0008
10	0.0020	0.0055	0.0015

## Data Availability

The data presented in this study are available on request from the corresponding author.

## Sample Availability

Samples of the compounds are available from the authors.
